# The Needs of the Future

**Published:** 1897-01

**Authors:** 


### Editorial.

### THE NEEDS OF THE FUTURE.

The opening of the New Year is apt to be as productive of thought as the passing away of the Old; but these reflections are as ancient, probably, as human intelligence, and may or may not lead to improved methods or a higher motive to action. The fact, nevertheless, remains that it is only by constant iteration upon ethical themes that man, in the concrete, develops into a higher standard. It is upon this constant repetition of what some may regard as platitudes that the moral life of civilization is founded, and the effect can only be observed by a careful analysis of the history of peoples as they have advanced slowly and painfully, step by step, to higher conceptions of duty to themselves and the world of which they form a part.

Professions are not exempt from this law of the moral life. They grow by silent and almost imperceptible accretions, here a little and there a little ; but ever the superstructure develops slowly into a compact body, worthy the respect and confidence of the community.

When dentistry is studied in its historical relations, the facts clearly show that it has been only by slow additions and through the work of a multitude of minds that it has reached present proportions, and what is true of dentistry is equally true of all the scientific work of the world.

The relation which each individual bears to the whole is well understood and requires no argument to demonstrate its force. Without combination nothing can be gained, and it is equally true that if the separate parts lack vitality the entire body will exhibit a corresponding weakness.

Accepting this view, what seems to be the duty of the individual in the coming time? As the individual is the integral part of the whole and represents it, it becomes the imperative duty of each to labor in his chosen department, that the sum of all the experience may represent the character of the entire body.

This is undoubtedly recognized by the few, and the work accomplished in the dental world, in the past few years, is in marked contrast to that which has preceded the present era; but the question arises, Is there not room for improvement among the masses that constitute the workers of this special calling?

The defect that must impress all readers of our literature is, that a large proportion of it is extremely weak from a scientific point of observation. The dental periodicals of the day teem with papers, some of them of lasting value, but the majority add nothing to the knowledge of the world upon the special subjects treated. The cause of this is not remote, but is to be attributed in part to the methods adopted by writers in general. The fault may, however, lie not so much with individuals as with societies. The latter must have essays to keep up the interest, and the demand is constant upon writers to supply the want. The temptation is an ever present one to comply. The paper is prepared, not from original investigations, but from the evolution of ideas in the individual brain, copiously illustrated by quotations from various authors. Assertions are dogmatically presented that have no other foundation than the crude conception of the person making them. It is no unusual thing to find writers boldly criticising the original investigations of others and, with a word, setting them aside as of no value. This not only leads to wrong thinking on the part of readers, but is a discouragement to original effort. The only true method is to either prove, by similar work, the truth of given facts or to antagonize them by similar labor.

It would, therefore, seem for the true development of the dentistry of the future that there should be an increase of scientific work. This can best be accomplished by societies demanding that the papers presented to them shall show originality, and by originality is meant not a mere dressing up of words into new combinations, but the evidence that the subject-matter comes fresh from the laboratory.

It may be argued that if this were adopted there would be nothing presented. This, unquestionably, is true of the present; but it does not, therefore, follow that no effort should be made to effect a change for the better. The amount of printed matter sent out to the world in the past, especially from this country, contains an amount of verbiage that is anything but creditable to dentistry. The weakness of the essay produces, as a necessary sequence, a similar result in the discussions which follow. Everything should be aimed towards thorough scientific work, and with an amount of proper stimulation brought to bear upon the new dentists of the period, a change must come, in the near future, which will make the old methods of procedure assume, to the new generation of workers, a grotesque character. The only true foundation, therefore, for a paper, on any subject, is scientific labor, or, a better phrasing, original investigation. It may not reach all of truth, but it will aid in clearing up disputed points, and will prove to be for the future investigator a mine of information for the final completion of his work.

The study of any subject presupposes a knowledge of all that has preceded it on that special line of investigation. There is, perhaps, nothing so annoying to the continuous reader as to find essayists stating supposed new things which the history of his profession reveals to him to be so old that their origin almost antedates professional memory, and yet it is well known that this is constantly being done by writers. To obviate this it should be one of the first duties of societies to establish professional libraries. It is not possible for every dentist to accumulate books, but it is possible for every local organization to build up a collection of infinite value as a library of reference. The importance of this is becoming apparent, and in the coming time these will be established in every professional centre.

The subject of organization is one that will be fully considered in the near future. The dental profession has followed precedent in this matter, and has taken the experience of the medical profession as its own. This has not worked altogether satisfactorily, and will never be in harmony with existing advanced ideas until these methods are changed. The discussion now in progress will, doubtless, produce good results, but there is evidently a feeling abroad to let things take their course. This is never a wise method. When a defect is discovered, the earlier measures are taken to remove it the better, and the many defects of dental organizations throughout the country are so apparent that the hour is ripe for a concentrated influence to be brought to bear to effect a change.

It is hoped the New Year will, at least, show an effort in the higher development of all organized bodies upon the lines suggested. Change cannot come suddenly; but if original investigation be encouraged and the writing of papers of no special value be discouraged, the gain will soon be apparent, and the dental profession in the United States will eventually be worthy of its name.

